# Genome-wide association analyses of agronomic traits and *Striga hermonthica* resistance in pearl millet

**DOI:** 10.1038/s41598-023-44046-1

**Published:** 2023-10-11

**Authors:** Armel Rouamba, Hussein Shimelis, Inoussa Drabo, Emmanuel Mrema, Christopher Ochieng Ojiewo, Learnmore Mwadzingeni, Abhishek Rathore

**Affiliations:** 1 African Centre for Crop Improvement, School of Agricultural, Earth and Environmental Sciences, University of KwaZulu-Natal, Private Bag X01, Scottsville, Pietermaritzburg, 3209 South Africa; 2 Institute of Environment and Agricultural Research, 01 BP 476, Ouagadougou, Burkina Faso; 3 Tanzania Agriculture Research Institute, Tumbi Center, P.O. Box 306, Tabora, Tanzania; 4 International Maize and Wheat Improvement Center, CIMMYT – ICRAF, House, United Nations Avenue, Gigiri, Nairobi, Kenya; 5 Seed Co Limited, 1 Shamwari Road, Stapleford, P.O. Box WGT 64 Westage, Harare, Zimbabwe; 6 Excellence in Breeding Platform (EiB), International Maize and Wheat Improvement Center, CIMMYT, Hyderabad, Telangana India

**Keywords:** Molecular biology, Plant sciences

## Abstract

Pearl millet (*Pennisetum glaucum* [L.] R. Br.) is a nutrient-dense, relatively drought-tolerant cereal crop cultivated in dry regions worldwide. The crop is under-researched, and its grain yield is low (< 0.8 tons ha^−1^) and stagnant in the major production regions, including Burkina Faso. The low productivity of pearl millet is mainly attributable to a lack of improved varieties, *Striga hermonthica* [*Sh*] infestation, downy mildew infection, and recurrent heat and drought stress. Developing high-yielding and *Striga-*resistant pearl millet varieties that satisfy the farmers’ and market needs requires the identification of yield-promoting genes linked to economic traits to facilitate marker-assisted selection and gene pyramiding. The objective of this study was to undertake genome-wide association analyses of agronomic traits and *Sh* resistance among 150 pearl millet genotypes to identify genetic markers for marker-assisted breeding and trait introgression. The pearl millet genotypes were phenotyped in *Sh* hotspot fields and screen house conditions. Twenty-nine million single nucleotide polymorphisms (SNPs) initially generated from 345 pearl millet genotypes were filtered, and 256 K SNPs were selected and used in the present study. Phenotypic data were collected on days to flowering, plant height, number of tillers, panicle length, panicle weight, thousand-grain weight, grain weight, number of emerged *Striga* and area under the *Striga* number progress curve (ASNPC). Agronomic and *Sh* parameters were subjected to combined analysis of variance, while genome-wide association analysis was performed on phenotypic and SNPs data. Significant differences (*P* < 0.001) were detected among the assessed pearl millet genotypes for *Sh* parameters and agronomic traits. Further, there were significant genotype by *Sh* interaction for the number of *Sh* and ASNPC. Twenty-eight SNPs were significantly associated with a low number of emerged *Sh* located on chromosomes 1, 2, 3, 4, 6, and 7. Four SNPs were associated with days-to-50%-flowering on chromosomes 3, 5, 6, and 7, while five were associated with panicle length on chromosomes 2, 3, and 4. Seven SNPs were linked to thousand-grain weight on chromosomes 2, 3, and 6. The putative SNP markers associated with a low number of emerged *Sh* and agronomic traits in the assessed genotypes are valuable genomic resources for accelerated breeding and variety deployment of pearl millet with *Sh* resistance and farmer- and market-preferred agronomic traits.

## Introduction

Pearl millet (*Pennisetum glaucum* [L.] R. Br., 2n = 2x = 14) is a highly nutritious and a key staple food crop in dry regions worldwide. It is the major crop of the Sahel region, including Burkina Faso^1^. In Africa, pearl millet is cultivated on an estimated area of 13.8 million hectares (ha), with an average yield of 0.7 tons ha^−1^^2^. In Burkina Faso, an estimated area of 1.2 million ha is devoted to pearl millet production. However, the mean yield of the crop in the country is low (< 0.81 tons ha^−1^), lesser than the global average of 0.9 tons ha^-1^^2^. The low grain yield in the farmers’ fields is attributable to various biotic and abiotic constraints, including the use of low-yielding landraces*, Striga hermonthica* (*Sh*) infestation, bird damage, insect pests, diseases, heat and drought stresses^3,4^.

*Striga hermonthica* (Del.) Benth. is the most significant biotic constraint to pearl millet production and productivity in Burkina Faso and yield losses vary between 40 and 55%^5,6^. The parasite infests several other major cereal crops, including rice (*Oryza glaberrima* Steudel and *O. Sativa* L.), maize (*Zea mays* L.), sorghum (*Sorghum bicolour* [L.] Moench), and fonio (*Digitaria exilis* [Kippist] Stapf)^7,8^. Farmers often abandon *Sh-*infested fields and switch from pearl millet to other non-host crops, reducing the crop’s overall production and economic value^9^. *Striga* is a highly prolific parasite in Burkina Faso attributed to the host crop being mostly grown in semi-arid parts of the Sahelian and Sudano-Sahelian zones, which are dominated by poor soil fertility, low and erratic rainfall, and high temperatures that favour germination, growth and spread of the weed^3^.

*Striga* control is difficult because each parasitic plant can quickly disperse and deposit thousands of seeds into the soil seedbank. Furthermore, *Striga* seeds can remain viable in the soils for more than 14 years^10^. *Striga hermonthica* is a major threat to food security, exacerbating hunger and poverty in many African countries^11,12^. Monetary losses ranging from 117 to 200 billion US$ is incurred annually due to crop damage, and increases by US $30 million annually^13^.

Several *Striga* control strategies are recommended, including hand weeding, mulching crop fields with biomass of the shea tree (*Vitellaria paradoxa* C.F. Gaertn.) as a bio-control agent, optimal fertilizer application, and soil moisture management^7,14^. These strategies improve the soil properties, promote crop growth and development, and retard germination and growth of *Striga*^15^. Herbicides are less effective in controlling the effect of the parasite after emergence, and they are unaffordable for smallholder farmers.

The use of *Striga*-resistant pearl millet varieties is the most sustainable and environment-friendly management option for smallholder farmers in semi-arid regions. Resistant cultivars support fewer *Striga* plants and yield higher^16,17^. However, with the paucity of locally adapted and *Sh*-resistant donor sources, breeding for *Striga* resistance in pearl millet is still challenging compared to other cereals^18–21^. In the past decade, intensive research on the interaction of *Striga* with the host at the molecular level has opened opportunities to develop new management strategies^22^. For instance, 154 candidate genes associated with *Sh* resistance traits were identified in maize^23^. Adewale et al.^24^ reported 13 associated markers with the *Sh* resistance trait in early maturing tropical white maize inbred lines.

Genome-wide association studies (GWAS) has been used in pearl millet for the identification of putative genes related to flowering time^25^, iron, zinc and protein content^26^, downy mildew resistance^27^ and *Sh* resistance^28^. Also, GWAS has been used in finger millet for the identification of genes associated with *Striga* resistance^29^ and grain nutritional contents^30^ and for genetic diversity analysis^31^. GWAS is a valuable genomic tool to identify quantitative trait loci (QTLs) linked to *Striga* resistance for marker-assisted selection. GWAS results depend on the genetic marker and its density, genetic composition and diversity of the test populations.

Genetic markers are landmarks on chromosomes that help pinpoint the location of genes of interest. They can be detected through morphological and molecular markers^9^. Genetic markers such as GRMZM2G077208, GRMZM2G164502, GRMZM2G018508, and GRMZM2G171986, located on chromosomes 3, 5, 7, and 9 were reportedly significantly associated with *Sh* count in tropical maize germplasm^32^. SNP markers are instrumental in the dissection of complex traits such as *Striga* resistance, and their association with the trait can be revealed through GWAS. Identification of genomic regions linked to *Striga* resistance in pearl millet breeding would speed up the development of *Striga*-resistant varieties. Genetic markers improve the efficiency of novel *Striga*-resistant genes introgression and pyramiding into high-yielding elite varieties through backcross method. Therefore, the objective of this study was to undertake genome-wide association analyses of agronomic traits and *Sh* resistance among 150 pearl millet genotypes to identify genetic markers for marker-assisted breeding and trait introgression.

## Results

### Phenotypic variations

Pearl millet genotypes differed significantly (*P* < 0.001) for days to 50% flowering (DTF), plant height (PH), number of tillers per plant (NT), panicle length (PCL), panicle weight (PWT), thousand-grain weight (TGW), and grain weight (GW) under *Sh* infestation. Genotypes differed significantly (*P* < 0.001) for the area under the *Striga* number progress curve (ASNPC). The genotype by *Striga* interaction was non-significant for the NT and ASNPC. The genotype by environment interaction differed significantly (*P* < 0.001) for days to 50% flowering (DTF), plant height (PH), number of tillers per plant (NT), panicle length (PCL), panicle weight (PWT), thousand-grain weight (TGW), and grain weight (GW) under *Sh* infestation (Table 1). Genotypes with missing data were excluded from the analysis.

**Table 1:** Mean squares and significant tests for pearl millet and *Striga* parameters when evaluating 137 genotypes with and without *Striga* infestation under the field and screen house environments in Burkina Faso.

Source of variation	DF	DTF	PH	NT	PCL
Replication	1	66.53 ns	3203.50***	20.93***	26.98 ns
Genotype	136	317.00***	2394.50***	4.12***	114.63***
*Striga*	1	389.51***	37,599.60***	156.73***	556.95***
Environment	1	5113.06***	84,488.00***	490.35***	2353.17***
Genotype x *Striga*	136	33.18***	526.60***	0.85 ns	12.13*
Genotype x Environment	136	65.94***	686.20***	1.24***	16.286***
Residual	416	21.80	341.70	0.78	8.94
Total	827				
Source of variation	DF	PWT	TGW	GW	ASNPC
Replication	1	131,169.00***	0.43 ns	11,016.00***	349.40 ns
Genotype	136	16,218.00***	14.04***	4953.00***	1627.00***
*Striga*	1	2,511,363.00***	73.23***	1,037,689.00***	120,730.90***
Environment	1	7,181,785.00***	250.04***	2,859,964.00***	88,892.80***
Genotype x *Striga*	136	4095.00 ns	4.03***	2375.00***	813.00 ns
Genotype x Environment	136	12,991.00***	3.74***	6013.00***	1688.70***
Residual	416	4421.00	2.46	1183.00	785.40
Total	827				

*, and *** = denote significant differences at 0.05, and 0.001 probability levels, respectively; ns = not significant; DF = degree of freedom; DTF = days to 50% flowering; PH = plant height at maturity (cm); NT = number of tillers; PCL = panicle length (cm); PWT = panicle weight (g); TGW = thousand-grain weight (g); GW = grain weight per plant (g); ASNPC = area under the *Striga* number progress curve.

**Figure 1:**
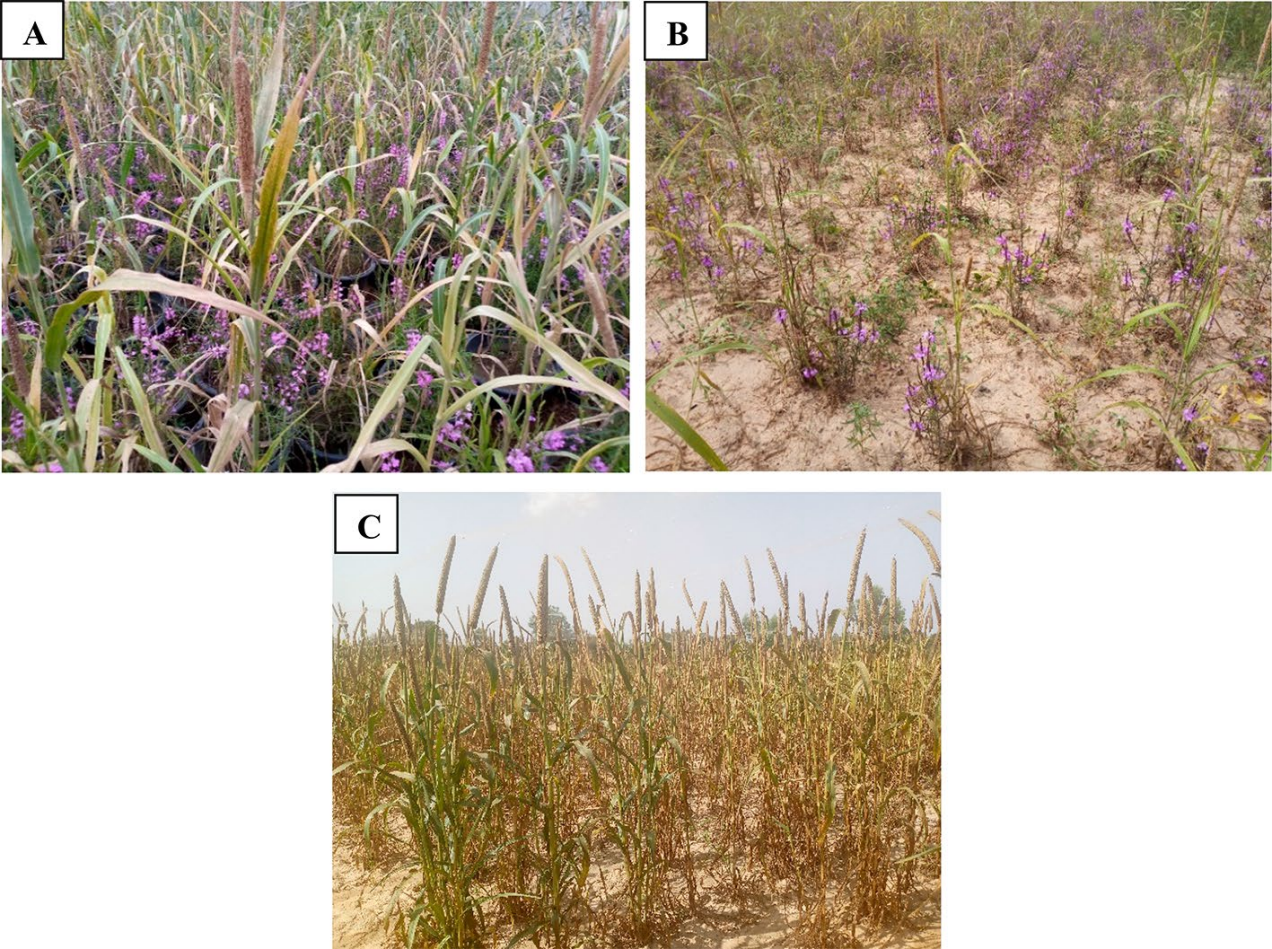
Pearl millet crop and *Striga* infestation in Burkina Faso. Note: *Striga hermonthica* infestation under screen house (**A**) and field (**B**) conditions. Photo C denotes *Striga* free field at the Didri site in Burkina Faso. (Photos supplied by Armel Rouamba).

Table 2 presented the best linear unbiased prediction means for the response of pearl millet genotypes evaluated under *Sh* infestation. The DTF and PH ranged from 60.13 to 64.95 days and 132.57 to 159.74 cm in the naturally-infested field and the screen house conditions, respectively. The TGW ranged from 7.60 to 9.03 g under *Striga* infestation in field and in screen house. Several emerged *Striga* were recorded during the second counting, particularly in the plastic pots (Fig. 1A) and the hotspot field (Fig. 1B) conditions compared to *Striga*-free field condition (Fig. 1C). Thousand-grain weight markedly reduced due to high *Striga* infestations compared to *Striga*-free field (Table 2).

**Table 2:** Best linear unbiased prediction means and standard error for 137 pearl millet genotypes evaluated in naturally *Sh* infested field and their genetic parameters.

Predicted value						
Trt/Trait	DTF	PH	PCL	TGW	SN1	SN2
GenInf	60.13	132.57	23.61	7.60	1.10	1.43
GenStr	64.95	153.06	19.42	8.97	0.87	0.88
Genotype	63.66	159.74	19.70	9.03		
Trial statistics						
SED (GenInf)	0.82	2.93	0.42	0.19	0.03	0.04
SED (GenStr)	0.84	2.99	0.41	0.21	0.03	0.03
SED (Genotype)	0.84	2.98	0.41	0.22		
H^2^ (bs)	87.48	84.46	92.42	75.07	28.61	18.17
LSD (5%)	ns	21.20	ns	1.92	ns	0.21
CV (%)	6.93	8.66	15.22	18.88	38.29	44.82

Trt = treatment; DTF = days to 50% flowering; PH = plant height at maturity (cm); PCL = panicle length (cm); TGW = thousand-grain weight (g); SN1 = the number of *Striga* counted 70 days after sowing in the *Striga*-infested field and 116 days after planting in the screen house; SN2 = *Striga* number counted 96 days after sowing in the *Striga*-infested field and 144 days after sowing in the screen house; GenInf = genotypes in naturally *Striga* infested field; GenStr = genotypes with *Striga* infestation in the screen house; SED = standard error of the mean difference; H^2^ (bs) = broad sense heritability; LSD = least significant difference; CV = coefficient of variation; ns = non-significant.

A low broad-sense heritability value was computed for the number of emerged *Sh* SN1 (18.17%) and SN2 (28.61%), while high heritability values ranging from 75.07 to 92.42% were recorded for the DTF, PCL, TGW, and PH (Table 2).

### Genome-wide association mapping

**Figure 2:**
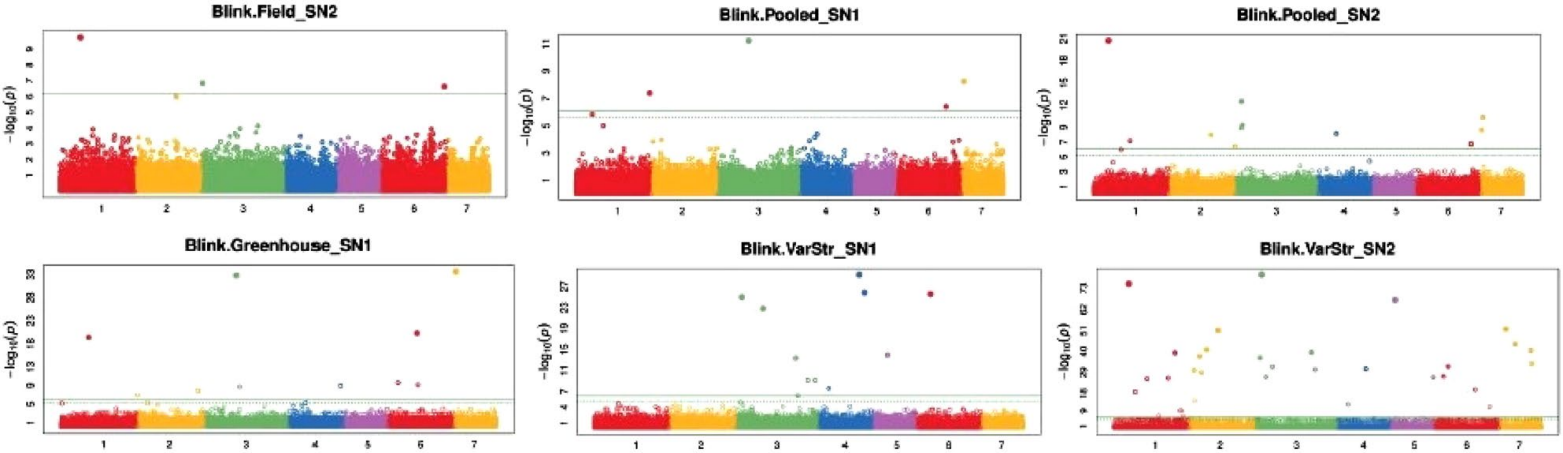
Manhattan plots showing associations between single nucleotide polymorphisms and SN1 and SN2 in naturally *Striga-*infested fields and greenhouse conditions. Single nucleotide polymorphisms were plotted on the x-axis according to their positions on each chromosome against association with *Sh*-related traits on the y-axis (−log 10 *p*-value). The top line indicates genome-wide significant threshold. Note: Blink.Field_SN2 = the Bayesian-information and linkage-disequilibrium iteratively nested keyway of *Striga* number counted 96 days after sowing in the *Striga*-infested field; Blink.Pooled_SN1 = the Bayesian-information and linkage-disequilibrium iteratively nested keyway of *Striga* number counted 70 days after sowing in the *Striga*-infested field and 116 days after planting in the greenhouse; Blink.Pooled_SN2 = the Bayesian-information and linkage-disequilibrium iteratively nested keyway of *Striga* number counted 96 days after sowing in the *Striga*-infested field and 144 days after sowing in the greenhouse; Blink.Greenhouse_SN1 = the Bayesian-information and linkage-disequilibrium iteratively nested keyway of *Striga* number counted 116 days after planting in the greenhouse, Blink.VarStr_SN1 = the Bayesian-information and linkage-disequilibrium iteratively nested keyway of *Striga* number counted 116 days after planting in the greenhouse for genotypes with *Striga* infestation; Blink.VarStr_SN2 = the Bayesian-information and linkage-disequilibrium iteratively nested keyway of *Striga* number counted 144 days after sowing in the greenhouse for genotypes with *Striga* infestation.

The number of emerged *Sh* count on the Manhattan plot is presented in Fig. 2. BLINK model analysis for *Sh* traits led to identifying candidate genetic regions associated with *Striga* resistance.

Twenty-eight SNP markers were significantly (*P* < 0,001) associated with *Sh* resistance located on chromosomes 1, 2, 3, 4, 6, and 7. Two SNPs, S3_113184999 and S3_113184999, were located on the same position and were associated with the number of *Sh* counted (Table 3). Three significant (*P* < 0,001) SNP markers, S1_75620319, S3_1159738, and S6_231436300, were associated with *Striga* resistance in the naturally *Striga*-infested field on chromosomes 1, 3, and 6. In the greenhouse, 10 significant SNPs were associated with *Sh*-resistance, while 15 SNPs were associated with *Sh*-resistance on chromosomes 1, 2, 3, 4, 6, and 7 in a pooled analysis.

**Table 3:** Markers significantly associated with *Striga* resistance traits in 150 pearl millet genotypes assessed in naturally infested fields and greenhouse conditions in Burkina Faso.

Trait	SNP	Chr	Position	P. value	MAF	AdjP	Annotation	Gene
Field_SN2	S1_75620319	1	75,620,319	1.80E-10	0.01369863	1.23E-05	Intergenic_region	GENE_CDS_1_75476337_75476480-GENE_CDS_1_75717949_75717961
Field_SN2	S3_1159738	3	1,159,738	1.41E-07	0.034246575	0.00480715	Intron_variant	Pgl_GLEAN_10031109
Field_SN2	S6_231436300	6	231,436,300	2.34E-07	0.020547945	0.005295382	Intergenic_region	GENE_CDS_6_231423518_231423673-GENE_CDS_6_231543192_231543410
Greenhouse_SN1	**S7_7890799**	7	**7,890,799**	2.13E-34	0.02739726	1.45E-29	Intergenic_region	GENE_CDS_7_7862940_7862984-GENE_CDS_7_7993524_7994057
Greenhouse_SN1	**S3_113184999**	3	**113,184,999**	1.37E-33	0.020547945	4.67E-29	Intergenic_region	GENE_CDS_3_113129296_113129642-GENE_CDS_3_113377327_113377432
Greenhouse_SN1	S6_108023970	6	108,023,970	4.21E-21	0.01369863	9.55E-17	Downstream_gene_variant	Pgl_GLEAN_10009280
Greenhouse_SN1	S1_99630014	1	99,630,014	3.61E-20	0.034246575	6.14E-16	Intergenic_region	GENE_CDS_1_99187249_99187566-GENE_CDS_1_99759745_99759760
Greenhouse_SN1	S6_39394185	6	39,394,185	2.43E-10	0.02739726	3.30E-06	Intergenic_region	GENE_CDS_6_39351328_39351639-GENE_CDS_6_39440122_39440890
Greenhouse_SN1	S6_111447163	6	111,447,163	7.07E-10	0.04109589	8.01E-06	Intergenic_region	GENE_CDS_6_111439201_111439539-GENE_CDS_6_111452367_111453077
Greenhouse_SN1	S4_182636703	4	182,636,703	1.24E-09	0.01369863	1.21E-05	Intron_variant	Pgl_GLEAN_10034091
Greenhouse_SN1	S3_126337466	3	126,337,466	2.16E-09	0.02739726	1.83E-05	Intergenic_region	GENE_CDS_3_126225889_126226120-GENE_CDS_3_126476234_126476446
Greenhouse_SN1	S2_218400344	2	218,400,344	1.35E-08	0.034246575	0.000101769	Intergenic_region	GENE_CDS_2_218366655_218366812-GENE_CDS_2_218413717_218413989
Greenhouse_SN1	S2_803192	2	803,192	1.10E-07	0.01369863	0.000748906	Synonymous_variant	Pgl_GLEAN_10017839
Pooled_SN1	**S3_113184999**	3	**113,184,999**	5.70E-12	0.020547945	3.88E-07	Intergenic_region	GENE_CDS_3_113129296_113129642-GENE_CDS_3_113377327_113377432
Pooled_SN1	**S7_7890799**	7	**7,890,799**	5.54E-09	0.02739726	0.000188415	Intergenic_region	GENE_CDS_7_7862940_7862984-GENE_CDS_7_7993524_7994057
Pooled_SN1	S1_270246408	1	270,246,408	4.10E-08	0.034246575	0.000929872	Upstream_gene_variant	Pgl_GLEAN_10038191
Pooled_SN1	S6_184283909	6	184,283,909	3.95E-07	0.020547945	0.006717724	Intergenic_region	GENE_CDS_6_184262794_184263538-GENE_CDS_6_184291509_184291562
Pooled_SN2	S1_54412075	1	54,412,075	1.86E-21	0.01369863	1.27E-16	Upstream_gene_variant	Pgl_GLEAN_10003483
Pooled_SN2	S3_22414758	3	22,414,758	3.15E-13	0.01369863	1.07E-08	Missense_variant	Pgl_GLEAN_10034999
Pooled_SN2	**S7_7890799**	7	**7,890,799**	4.50E-11	0.02739726	1.02E-06	Intergenic_region	GENE_CDS_7_7862940_7862984-GENE_CDS_7_7993524_7994057
Pooled_SN2	S3_26238337	3	26,238,337	4.89E-10	0.01369863	8.31E-06	Intergenic_region	GENE_CDS_3_26144223_26144501-GENE_CDS_3_26273998_26275158
Pooled_SN2	S3_22104285	3	22,104,285	1.24E-09	0.01369863	1.69E-05	Upstream_gene_variant	Pgl_GLEAN_10034978
Pooled_SN2	S7_2011566	7	2,011,566	2.33E-09	0.034246575	2.64E-05	Synonymous_variant	Pgl_GLEAN_10022900
Pooled_SN2	S4_68863333	4	68,863,333	7.15E-09	0.01369863	6.95E-05	Downstream_gene_variant	Pgl_GLEAN_10029552
Pooled_SN2	S2_152870927	2	152,870,927	1.09E-08	0.01369863	9.25E-05	Intergenic_region	GENE_CDS_2_152669342_152669906-GENE_CDS_2_153313889_153313993
Pooled_SN2	S1_133683128	1	133,683,128	6.95E-08	0.04109589	0.000525221	Intergenic_region	GENE_CDS_1_133517524_133517793-GENE_CDS_1_134364799_134365161
Pooled_SN2	S6_204391395	6	204,391,395	1.79E-07	0.01369863	0.001214075	Intergenic_region	GENE_CDS_6_204203637_204204055-GENE_CDS_6_204398055_204398642
Pooled_SN2	S2_242049249	2	242,049,249	3.82E-07	0.123287671	0.002360813	Upstream_gene_variant	Pgl_GLEAN_10018078

SNP = single nucleotide polymorphism, Chr. = chromosome, MAF = minor allele frequency, AdjP = false discovery rate adjusted P-values, SN1 = the number of *Striga* counted 70 days after sowing in the *Striga*-infested field and 116 days after planting in the screen house; SN2 = *Striga* number counted 96 days after sowing in the *Striga*-infested field and 144 days after sowing in the screen house.

### Agronomic traits

**Figure 3:**
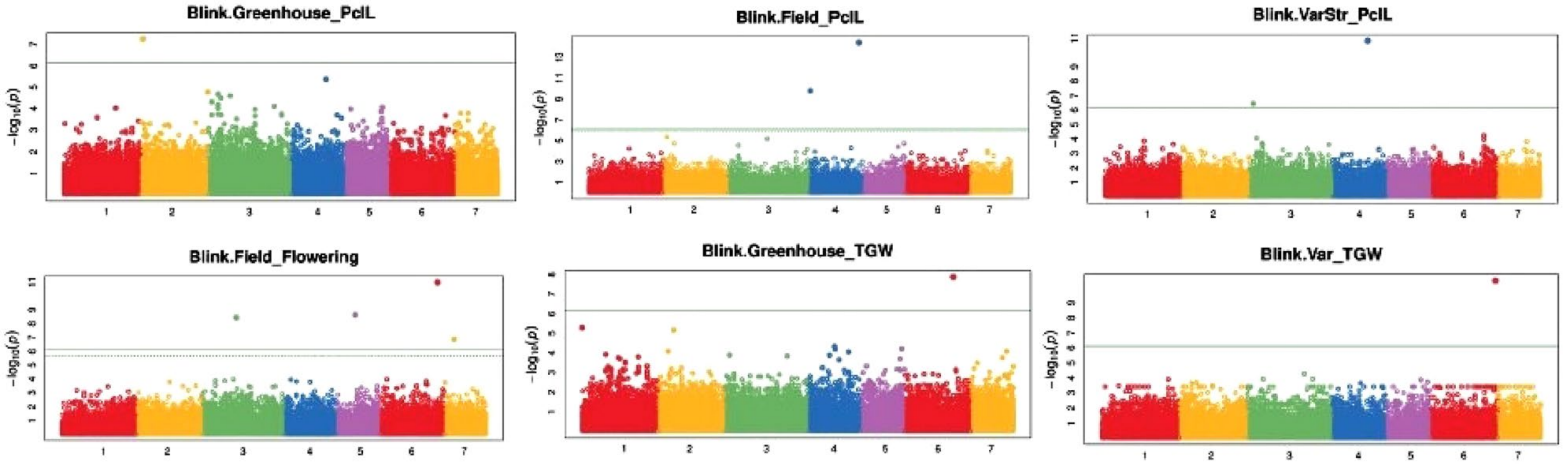
Manhattan plots showing associations between single nucleotide polymorphisms and panicle length, flowering time and thousand-grain weight under *Sh* conditions in naturally *Striga* infested field and greenhouse. Single nucleotide polymorphisms were plotted on the x-axis according to their positions on each chromosome against association with pearl millet-related traits on the y-axis (−log 10 *p*-value). The top line indicates genome-wide significant threshold. Note: Blink.Greenhouse_PclL = the Bayesian-information and linkage-disequilibrium iteratively nested keyway of panicle length in screen house; Blink.Field_PclL = the Bayesian-information and linkage-disequilibrium iteratively nested keyway of panicle length in the field; Blink.VarStr_PclL = the Bayesian-information and linkage-disequilibrium iteratively nested keyway of panicle length for genotypes with *Striga* infestation; Blink.Greenhouse_TGW = the Bayesian-information and linkage-disequilibrium iteratively nested keyway of thousand-grain weight in the screen house, Blink.Var_TGW = the Bayesian-information and linkage-disequilibrium iteratively nested keyway of thousand-grain weight for genotypes in the screen house.

BLINK model analysis of pearl millet agronomic traits under *Sh* infestation identified candidate genetic regions associated with DTF, PCL, and TGW (Fig. 3). Eleven SNP markers were associated with the assessed pearl millet phenotypic traits. Four SNPs were associated with DTF on chromosomes 3, 5, 6, and 7 in the naturally *Striga*-infested field; five with PCL on chromosomes 2, 3, and 4 with which two in the naturally *Striga*-infested field and three in the screen house; and two with TGW on chromosome 6 in the screen house (Table 4).

**Table 4:** Markers significantly associated with agronomic traits in 150 pearl millet genotypes assessed in naturally *Striga*-infested fields and screen house conditions in Burkina Faso.

Trait	SNP	Chr	Position	P. value	MAF	AdjP	Annotation	Gene
Field_Flowering	S6_214124725	6	214,124,725	1.05E-11	0.02739726	7.13E-07	Intergenic_region	GENE_CDS_6_214064703_214065150-GENE_CDS_6_214144811_214144961
Field_Flowering	S5_70437618	5	70,437,618	2.36E-09	0.04109589	8.03E-05	Intron_variant	Pgl_GLEAN_10010062
Field_Flowering	S3_124462289	3	124,462,289	3.65E-09	0.01369863	8.26E-05	Intergenic_region	GENE_CDS_3_124448276_124448524-GENE_CDS_3_124546255_124546494
Field_Flowering	S7_34887211	7	34,887,211	1.34E-07	0.034246575	0.002276945	Intron_variant	Pgl_GLEAN_10030410
Field_PclL	S4_178741335	4	178,741,335	3.80E-15	0.02739726	2.58E-10	Synonymous_variant	Pgl_GLEAN_10011399
Field_PclL	S4_4389968	4	4,389,968	1.65E-10	0.01369863	5.61E-06	Downstream_gene_variant	Pgl_GLEAN_10014324
Greenhouse_PclL	S2_9048490	2	9,048,490	5.77E-08	0.047945205	0.003923655	Intron_variant	Pgl_GLEAN_10013735
Greenhouse_TGW	S6_176707138	6	176,707,138	1.36E-08	0.02739726	0.000922491	Intergenic_region	GENE_CDS_6_176509156_176509570-GENE_CDS_6_176832456_176832571
VarStr_PclL	S4_125228939	4	125,228,939	1.61E-11	0.01369863	1.10E-06	Downstream_gene_variant	Pgl_GLEAN_10035535
VarStr_PclL	S3_14571318	3	14,571,318	3.65E-07	0.205479452	0.012407417	Upstream_gene_variant	Pgl_GLEAN_10018534
Var_TGW	S6_232020017	6	232,020,017	3.26E-11	0.020547945	2.22E-06	Downstream_gene_variant	Pgl_GLEAN_10024656

SNP = single nucleotide polymorphism, Chr. = chromosome, MAF = minor allele frequency, AdjP = false discovery rate adjusted P.values.

## Discussion

The assessed genotypes exhibited significant differences in agronomic traits and *Sh* parameters (Table 1), suggesting substantial genetic variation for selection. The results allowed marker-trait association analysis to be valuable for current and future selection and new variety design and commercialisation. A significant genotype by the *Sh* interaction effect existed (Table 1), revealing the potential existence of genes controlling *Sh* resistance among the populations. This concurs with the deductions made by Mrema et al.^8^ and Shayanowako et al.^33^, who reported significant variation and differential genotypic responses to *Sh* infestation among sorghum and maize genotypes, in that order. Thus, the population set evaluated in the current study was suitable for marker-trait association analyses for *Sh* resistance and economic traits.

Reduced DTF and PH were recorded on pearl millet genotypes under *Striga* infestation (Table 2), indicating the negative impact of the parasite on the measured traits. These findings concur with reports by Ransom and Odhiambo^34^. Wilson et al.^18^ reported a negative correlation between the number of emerged *Sh* and DTF in pearl millet. Similarly, a reduction of PH by 28% under *Sh* infestation was reported in pearl millet by Graves et al.^35^. Badu-Apraku^36^ reported a negative correlation between *Striga* damage rating and DTF in maize in Sub-Saharan Africa. Poor crop growth and subsequent low productivity result from the *Striga* plant attachment and siphoning of nutrients from the host plant's roots.

The recorded TGW of 7.60 to 9.03 g in the present study (Table 2) aligns with the 6.9 to 12.9 g reported by Kanatti et al.^37^. The relatively high ranges of values in the current results are probably attributed to the large grain size, which increased the yield of pearl millet^37,38^. The broad-sense heritability (H^2^) for agronomic traits ranged from 75.07 to 92.42% and 18.17 to 28.61% for emerged *Sh* count (Table 2). The high broad-sense heritability estimates of 75.07 to 92.42% computed for DTF, PH, PCL, and TGW indicated that the traits are mainly governed by genes with limited influence by the test environment^39^. Traits with high heritability are easy to select and improve using marker-assisted selections and pyramiding in a desirable genetic background. The lower broad-sense heritability estimates of 18.17 and 28.61% for the number of emerged *Striga* (SN1 and SN2) (Table 2) suggests that the genetic variation was small and genetic gain for those traits will be slow because both genetic and phenotypic constituents of the genotypes are affected by *Striga* infestation stress^39^. Robert^40^ and Kaewchumnong and Price^41^ reported a low heritability estimate for *Striga* resistance-related traits in sorghum and rice, respectively.

The seven pearl millet chromosomes harbour several genes (Table 4) conditioning *Striga* resistance and agronomic traits. Each chromosome had at least two significant marker-trait associations in the present study. After successful validation, the 28 significant SNP markers associated with *Sh* emergence on chromosomes 1, 2, 3, 4, 6, and 7 (Table 3) can be used for marker-assisted selection and trait introgression to improve *Sh* resistance in pearl millet. Dawud^28^ identified 16 SNP markers associated with the area under the *Striga* number progress curve on chromosomes 2, 3, 4, 5, and 7 in pearl millet. The findings confirm that the chromosomes harbour some beneficial alleles influencing *Sh* resistance. Dawud^28^ reported significant gene markers related to *Striga* resistance on chromosomes 1, 2, 3, and 5 in pearl millet. Markers associated with *Striga* resistance traits have also been reported in sorghum and maize^44,45^. The two SNP makers located adjacent to each other and associated with the low number of *Sh* count could be tightly linked and co-segregating. Hence, the respective candidate genes can be selected and introgressed simultaneously^24^. Identifying genetic markers associated with agronomic traits will facilitate marker-assisted breeding in pearl millet (Fig. 3). Using SSR markers, Kannan et al.^46^ detected significant markers associated with pearl millet panicle length and thousand-grain weight in *Striga*-free conditions. In maize, some quantitative trait loci associated with grain yield and ear aspect have been reported by Stanley et al.^47^ and Badu-Apraku et al.^23^. Dawud^28^ also reported Significant SNP markers related to the number of tillers in pearl millet. This study identified 28 SNP markers associated with low *Sh* emergence on chromosomes 1, 2, 3, 4, 6, and 7, involving genetic analysis of 150 genetically diverse pearl millet genotypes in Burkina Faso (Fig. 3). The candidate markers and genotypes are novel genomic and genetic resources for *Striga* resistance breeding programs in the country and elsewhere.

## Materials and methods

### Study sites

A field experiment was conducted in the 2019/20 main growing season in a naturally infested *Striga* hotspot field at the Didri site in Burkina Faso, and a screen house evaluation was conducted at the main station of the Institute of Environment and Agricultural Research (INERA) in the offseason of 2020/21. The Didri site is located at 12° 12′ 15" N and 1° 14′ 13" W and is a hotspot site for *Sh* affecting pearl millet, maize and sorghum crops. The site received an annual rainfall of 748.5 mm for 46 days during the 2019/20 rainy season and has sandy soils. The INERA site is located at 12°28/27 N and 1°33/31W.

### Plant materials

The study used 148 pearl millet genotypes collected from the International Crop Research Institute for the Semi-arid Tropics (ICRISAT) in Niger and two elite breeding lines from INERA/Burkina Faso. The descriptions of the test genotypes are summarised in Table 5. The pearl millet genotypes acquired from ICRISAT are part of the pearl millet germplasm association panel (PMiGAP) comprising 250 inbred lines representing cultivated germplasm from Africa and Asia. They are included in the present study to identify unique genetic resources with unique agronomic and farmers’ preferred traits, and because of their wide genetic diversity.

**Table 5:** Description of the pearl millet genotypes used in the study.

E.N	Genotype code	Pedigree or name	Source	Presumed *Striga* resistance	E.N	Genotype code	Pedigree	Source	Presumed *Striga* resistance
1	IP-2058	Z 42	ICRISAT	S	39	IP-7633	S 195	ICRISAT	S
2	IP-3098	–	ICRISAT	R	40	IP-7886		ICRISAT	S
3	IP-3110	–	ICRISAT	S	41	IP-7910	D 89 C-1–1	ICRISAT	S
4	IP-3122	–	ICRISAT	S	42	IP-7922	IP 5238–2; D 175 C-2–2	ICRISAT	S
5	IP-3125	–	ICRISAT	S	43	IP-7942	IP 5452–1; P 2742–1	ICRISAT	S
6	IP-3175	–	ICRISAT	S	44	IP-7952	IP 6578–1; Kolala local 7–1	ICRISAT	S
7	IP-3389	–	ICRISAT	S	45	IP-7953	IP 6191–1; P 87–1	ICRISAT	S
8	IP-3564	–	ICRISAT	S	46	IP-7967	IP 6342–1; P 337–2	ICRISAT	S
9	IP-3593	–	ICRISAT	S	47	IP-8002	37 K-1–1	ICRISAT	S
10	IP-3732	–	ICRISAT	S	48	IP-8129	GS 112	ICRISAT	S
11	IP-3757	–	ICRISAT	S	49	IP-8166	GS 148	ICRISAT	S
12	IP-3865	–	ICRISAT	S	50	IP-8172	GS 154	ICRISAT	S
13	IP-3890	–	ICRISAT	S	51	IP-8174	GS 156	ICRISAT	S
14	IP-4378	–	ICRISAT	S	52	IP-8181	IP 338–1	ICRISAT	S
15	IP-4927	Souna D2	ICRISAT	S	53	IP-8182	IP 406-B-1	ICRISAT	S
16	IP-4974	700,111	ICRISAT	S	54	IP-8187	IP 2695–1	ICRISAT	S
17	IP-5031	700,482	ICRISAT	S	55	IP-8210	IP 1739 L-1	ICRISAT	S
18	IP-5131	D 235	ICRISAT	S	56	IP-8276	IP 2130–1/CG 51	ICRISAT	S
19	IP-5272	D 258	ICRISAT	S	57	IP-8280	Souna 57–1	ICRISAT	S
20	IP-5438	P 2727	ICRISAT	S	58	IP-8294	IP 6132–1; P 24–2	ICRISAT	S
21	IP-5695	45–327	ICRISAT	S	59	IP-8426	SDN 496–1	ICRISAT	S
22	IP-5713	45–349	ICRISAT	S	60	IP-8761	–	ICRISAT	S
23	IP-5816	P 1407/S1.45	ICRISAT	S	61	IP-8767	–	ICRISAT	S
24	IP-5900	P 1505/S1.228	ICRISAT	S	62	IP-8786	–	ICRISAT	S
25	IP-5923	P 1531–1/S1.293	ICRISAT	S	63	IP-8863	–	ICRISAT	S
26	IP-6099	P 932	ICRISAT	S	64	IP-8949	P 3254; PL 73	ICRISAT	S
27	IP-6103	P 939	ICRISAT	S	65	IP-9242	Sanio 35	ICRISAT	R
28	IP-6111	P 947	ICRISAT	S	66	IP-9347	–	ICRISAT	S
29	IP-6112	P 949	ICRISAT	R	67	IP-9651	PI 286,865	ICRISAT	S
30	IP-6584	–	ICRISAT	S	68	IP-9692	PI 286,979	ICRISAT	S
31	IP-6682	–	ICRISAT	S	69	IP-9710	PI 287,043	ICRISAT	S
32	IP-6745	–	ICRISAT	S	70	IP-9854	Acc 50–1	ICRISAT	S
33	IP-6769	–	ICRISAT	S	71	IP-9969	1769	ICRISAT	S
34	IP-6882	Acc 124	ICRISAT	S	72	IP-10085	P 5439	ICRISAT	S
35	IP-6891	Acc 144	ICRISAT	S	73	IP-10339	–	ICRISAT	S
36	IP-6892	Acc 147	ICRISAT	S	74	P-10471	–	ICRISAT	S
37	IP-7470	–	ICRISAT	S	75	IP-10486	–	ICRISAT	S
38	IP-7536	K 46	ICRISAT	S	76	IP-10488	–	ICRISAT	S
**E.N**	**Genotype code**	**Pedigree**	**Source**	**Presumed *Striga* resistance**	**E.N**	**Genotype codes**	**Pedigree**	**Source**	**Presumed *Striga* resistance**
77	IP-10579	CMM 410	ICRISAT	R	115	IP-15872	P 15	ICRISAT	S
78	IP-10705	CMM 540	ICRISAT	S	116	IP-15917	NPT 1	ICRISAT	S
79	IP-10820	Acc 615	ICRISAT	S	117	IP-16289	–	ICRISAT	S
80	IP-10953	BM 8	ICRISAT	S	118	IP-16403	–	ICRISAT	S
81	IP-10964	–	ICRISAT	S	119	IP-17099	–	ICRISAT	S
82	IP-11310	CVP 152	ICRISAT	S	120	IP-17150	–	ICRISAT	S
83	IP-11346	CVP 278	ICRISAT	S	121	IP-17554	–	ICRISAT	S
84	IP-11353	CVP 298	ICRISAT	S	122	IP-17611	–	ICRISAT	S
85	IP-11358	CVP 311	ICRISAT	R	123	IP-17690	–	ICRISAT	S
86	IP-11577	P 6041	ICRISAT	S	124	IP-18062	–	ICRISAT	S
87	IP-11593	P 6062	ICRISAT	S	125	IP-18147	–	ICRISAT	S
88	IP-11670	Millet 199	ICRISAT	S	126	IP-18246	–	ICRISAT	S
89	IP-11677	100	ICRISAT	S	127	IP-18293	BLP 1	ICRISAT	S
90	IP-11763	Arnold 2131	ICRISAT	S	128	IP-18500	–	ICRISAT	S
91	IP-11765	Arnold 2141	ICRISAT	S	129	IP-18621	–	ICRISAT	S
92	IP-12128	–	ICRISAT	S	130	IP-19334	–	ICRISAT	S
93	IP-12138	–	ICRISAT	S	131	IP-19361	–	ICRISAT	S
94	IP-12298	–	ICRISAT	S	132	IP-19386	–	ICRISAT	S
95	IP-12322	–	ICRISAT	S	133	IP-19388	–	ICRISAT	S
96	IP-12364	–	ICRISAT	S	134	IP-19612	C 90–119	ICRISAT	S
97	IP-12395	JM 4615	ICRISAT	S	135	IP-19613	C 90–120	ICRISAT	S
98	IP-12840	–	ICRISAT	S	136	IP-19626	C 90–133	ICRISAT	S
99	IP-12967	–	ICRISAT	S	137	IP-21020	–	ICRISAT	S
100	IP-13016	P 565–1	ICRISAT	S	138	IP-21169	P 1449–3	ICRISAT	S
101	IP-13154	Maiwa local 2–1	ICRISAT	S	139	IP-21206	D 332/1/2–2	ICRISAT	S
102	IP-13180	No. 2–1	ICRISAT	T	140	IP-21517	–	ICRISAT	S
103	IP-13324	Acc 9–1	ICRISAT	S	141	IP-22419	ICML 1; ICMPE 13–6-27	ICRISAT	S
104	IP-13344	Acc 736–1	ICRISAT	S	142	IP-22420	ICML 2; ICMPE 13–6-30	ICRISAT	S
105	IP-13363	–	ICRISAT	S	143	IP-22423	ICML 5; SSC FS 252-S-4	ICRISAT	S
106	IP-13459	–	ICRISAT	S	144	IP-22424	ICML 6; ICI 7517-S-1	ICRISAT	S
107	IP-13817	CVP 230	ICRISAT	S	145	IP-22455	ICMP 85,410	ICRISAT	S
108	IP-13964	–	ICRISAT	S	146	IP-22494	ARD 282 (133)	ICRISAT	S
109	IP-13971	–	ICRISAT	S	147	IP-21142	Tifton 186	ICRISAT	S
110	IP-14210	–	ICRISAT	S	148	SOSAT-C88		ICRISAT	C
111	IP-14624	–	ICRISAT	S	149	IKMP5-S4-41	IKMP5	INERA	S
112	IP-15320	–	ICRISAT	S	150	MISARI 1-S4-27	MISARI 1	INERA	S
113	IP-15533		139	ICRISAT	S				
114	IP-15857		–	ICRISAT	C				

E.N. = entry number; ICRISAT = International Crop Research Institute for the Semi-Arid Tropics; INERA = Institute of Environment and Agricultural Research/Burkina Faso; C = check; R = resistance; T = tolerance; S = susceptible,—= denote data not available.

### Experimental design and trial management

The field and greenhouse experiments were laid out using a 10 × 15 alpha lattice design with two replicates. In the greenhouse, 5L plastic pots were used and filled with a soil medium composed of clay, sand, and organic manure at a ratio of 2:1:1 respectively. Two weeks before planting, each pot was infested with a scoop of sand mixed with 0.05 g of 1-year-old *Sh* seed collected from farmers' fields in Burkina Faso. Pearl millet seeds in the naturally *Striga*-infested field (hereafter designated as GenInf), were sown during the main crop growing season from June to October 2019. Genotypes were established in 4.2 m long rows spaced at an inter-row spacing of 160 cm and intra-row spacing of 60 cm, providing a total plot size of 6.72 m^2^ per genotype. Four seeds were initially sown per hill and later thinned to one plant two weeks after planting. A total of three plants was selected randomly from the middle of the experimental unit and tagged for agronomic data collection. In the greenhouse, one healthy and vigorous plant was grown per pot for the test genotypes with *Sh* (hereafter denoted as GenStr), and genotypes without *Sh* (hereafter referred to as control) treatments. Standard agronomic practices recommended for pearl millet production were followed. Experimental units were fertilized using nitrogen, phosphorus and potassium (NPK: 14:23:14) and applied as a microdose of 3 g per hill 15 days after planting. Hand weeding was routinely done after the first hoeing to remove all other weeds except *Striga*.

### Data collection

#### Phenotypic data

The following agronomic parameters were collected from pearl millet: days-to-50%-flowering (DTF) were recorded as the days when 50% of the plants in each plot had intruded stigma. Plant height (PH) was measured in cm from the base to the top of the panicle of the main tiller. The number of tillers per plant (NT) was recorded by counting the number of tillers with panicles for the tagged plants. Panicle length (PCL) was measured in cm from the base to the top of the main tiller panicle. Panicle weight (PWT) was recorded in grams by weighing the harvested panicles for each entry after 14 days of sun-dry, and thousand-grain weight (TGW) was determined in grams by weighing one thousand-grain for each of the entries. Grain weight per plant (GW) was determined in grams by weighing the grain after threshing and dividing it by the number of harvested plants for each plot.

For *Striga* parameters, the number of emerged *Sh* plants per plot were recorded at 70 and 96 days after sowing in the naturally infested field for each row, excluding the borders. The number of emerged *Sh* plants were counted per pot 116 and 144 days after sowing in the greenhouse. The area under the *Striga* Number Progress Curve (ASNPC) was computed using the successive *Striga* counts according to Haussmann et al.^48^ as follows:

$$ASNPC=\sum_{i=0}^{n-1}\left[\frac{{Y}_{i}+{Y}_{\left(i+1\right)}}{2}\right]\left({t}_{i+1}-{t}_{i}\right)$$

where n is the number of *Striga* assessment dates, *Y*_*i*_ is the *Striga* count at the *i*th assessment date, *Y*_*(i*+*1)*_ is the *Striga* count at the *i*th plus 1 assessment date, *t*_*i*_ is the number of days after planting (DAP) at the *i*th assessment date, *t*_*(i*+*1)*_ is DAP at the *i*th plus 1 assessment date.

#### Phenotypic data analysis

Both the crop and *Striga* data were subjected to analysis of variance using GenStat 19th Edition (http://www.genstat.co.uk). Homogeneity of variance test was done for each site using the Bartlett^49^ procedure before combined analyses. The treatment, genotype, and genotype × treatment interaction significance tests were computed using GenStat. The Best Linear Unbiased Prediction (BLUP) was calculated according to Haslett and Puntanen^50^ to predict the accuracy and to aid selection. The area under the *Striga* number progress curve was drawn using R. ASReml-R Version 4 was used to fit the linear mixed models using Residual Maximum Likelihood (REML) in R^51^.

#### Genotyping and GWAS analysis

To assemble the pearl millet genome, whole genome shotgun (WGS) and bacterial artificial chromosome (BAC) sequencing were used. Ten small inserts (of ~ 170, 250, 500 and 800 bp) and 13 large inserts (of ~ 2, 5, 10, 20 and 40 kb) WGS libraries were constructed using Tift 23D2B1-P1-P510 genotype. These libraries were sequenced on the Illumina HiSeq 2000, and 520 Gb of sequence data, representing 296 × genome coverage. Two BAC libraries, with an average insert size of ~ 120 kb, were constructed from Tift 23D2B1-P1-P5 using EcoRI and HindIII. Nine hundred seventy-two Gb of sequence data were generated from 100,608 BAC clones at ~ 80 × genome coverage. In brief, 1.49 Tb of sequence data, after stringent filtering and correction steps, were assembled into 1.58 Gb of contigs and 1.82 Gb of scaffolds (https://doi.org/10.1038/nbt.3943). A raw marker set consisting of 29 million SNPs generated from 345 pearl millet genotypes from which 148 genotypes used in the study was sourced from the Nature Paper Pearl Millet (https://doi.org/10.1038/nbt.3943) and filtered using Tassel v4.2 for site coverage of 90%, minor allele frequency of 0.1, taxa coverage of 30% and maximum heterozygosity of 50%. The resulting final set of variants contained 256 K SNPs was used for the current analysis. Phenotypic data collected from 150 genotypes were available for marker-trait association analysis. Principal component analysis (PCA) was calculated, and the resulting eigenvalue (7) was used for genome-wide association analysis (GWAS) following multiple methods procedures generated from GAPIT v3.0^52^. The Bayesian-information and linkage-disequilibrium iteratively nested keyway (BLINK) software was used to determine the significant variations among pearl millet and *Sh* traits owing to its ability to produce fewer false positives and more true positives than the GWAS method, FarmCPU^42^. Liu et al.^43^ reported the power of BLINK to outperform FarmCPU relative to statistical capabilities vs False Discovery Rate (FDR) and statistical power vs type I error.

## Conclusion

The current study detected significant genetic variability and markers for *Sh* resistance and agronomic traits through GWAS involving 150 pearl millet genotypes in Burkina Faso. There were significant genotypes by *Sh* interaction for assessed agronomic traits, the number of *Sh* and ASNPC. Twenty-eight SNPs were associated with *Sh* traits on chromosomes 1, 2, 3, 4, 6, and 7. SNPs markers associated with DTF, PCL, and TGW were located on chromosomes 2, 5, 6, and 7; chromosomes 2, 3, and 4; and chromosome 6, respectively. After successful validation, the new markers would be deployed for marker-assisted breeding emphasising the above agronomic traits and *Sh* resistance in pearl millet in Burkina Faso or related agro-ecologies.

## Data Availability

The datasets generated and/or analysed during the current study are available in the [OAR@ICRISAT] repository, [https://cegresources.icrisat.org/data_public/PM_SNPs/SNP_calls/].
