# Author Correction: Genome-wide association analyses of agronomic traits and *Striga hermonthica* resistance in pearl millet

**DOI:** 10.1038/s41598-023-45365-z

**Published:** 2023-10-25

**Authors:** Armel Rouamba, Hussein Shimelis, Inoussa Drabo, Emmanuel Mrema, Christopher Ochieng Ojiewo, Learnmore Mwadzingeni, Abhishek Rathore

**Affiliations:** 1 African Centre for Crop Improvement, School of Agricultural, Earth and Environmental Sciences, University of KwaZulu-Natal, Private Bag X01, Scottsville, Pietermaritzburg, 3209 South Africa; 2 Institute of Environment and Agricultural Research, 01 BP 476, Ouagadougou, Burkina Faso; 3 Tanzania Agriculture Research Institute, Tumbi Center, P.O. Box 306, Tabora, Tanzania; 4 International Maize and Wheat Improvement Center, CIMMYT – ICRAF, House, United Nations Avenue, Gigiri, Nairobi, Kenya; 5 Seed Co Limited, 1 Shamwari Road, Stapleford, P.O. Box WGT 64 Westage, Harare, Zimbabwe; 6 Excellence in Breeding Platform (EiB), International Maize and Wheat Improvement Center, CIMMYT, Hyderabad, Telangana India

Correction to: *Scientific Reports* 10.1038/s41598-023-44046-1, published online 11 October 2023

The Acknowledgements section in the original version of this Article was incomplete,

“The research was funded by the International Crops Research Institute for the Semi-Arid Tropics (ICRISAT) through ‘Harnessing Opportunities for Productivity Enhancement (HOPE II) for Sorghum and Millets in sub-Saharan Africa’ project (OPP1198373), funded by the Bill & Melinda Gates Foundation (BMGF).”

now reads:

“The research was funded by the International Crops Research Institute for the Semi-Arid Tropics (ICRISAT) through ‘Harnessing Opportunities for Productivity Enhancement (HOPE II) for Sorghum and Millets in sub-Saharan Africa and CIMMYT through the Accelerated Varietal Improvement and Seed delivery of legumes and cereals in Africa (AVISA) (grant number OPP1198373), funded by the Bill & Melinda Gates Foundation (BMGF).”

The original Article has been corrected.

